# Differentiation between Ripening Stages of Iberian Dry-Cured Ham According to the Free Amino Acids Content

**DOI:** 10.3390/foods9010082

**Published:** 2020-01-12

**Authors:** Ángela Alcazar Rueda, José Marcos Jurado, Fernando de Pablos, Manuel León-Camacho

**Affiliations:** 1 Department of Analytical Chemistry, Faculty of Chemistry, University of Seville, calle Profesor García González 1, E-41012 Seville, Spainjmjurado@us.es (J.M.J.); fpablos@us.es (F.d.P.); 2 Lipid Characterization and Quality Department. Instituto de la Grasa (C.S.I.C.), Ctra. Utrera km 1, Campus Universitario Pablo de Olavide, Edificio 46, E-41013 Seville, Spain

**Keywords:** free amino acids, gas chromatography, mass spectrometry, Iberian dry-cured ham, food authentication, chemometrics, pattern recognition

## Abstract

In this paper, the differentiation of three ripening stages, postsalting, drying, and cellar, of Iberian dry-cured ham has been carried out according to their free amino acids contents. Eighteen L-amino acids, alanine, 2-aminobutanoic acid, aspartic acid, cysteine, glutamine, glycine, histidine, hydroxyproline, isoleucine, leucine, lysine, methionine, phenylalanine, proline, serine, threonine, tyrosine, and valine have been determined by gas chromatography with derivatization with N,O-*bis*(trimethylsilyl)-trifluoroacetamide. Gas chromatography–mass spectrometry was used to confirm the presence of the eighteen amino acids in the ham samples, and gas chromatography using a DB-17HT column and flame ionization detector was used for quantitative determination. Extraction with a mixture methanol-acetonitrile has been carried out, achieving recoveries in the range 52–164%. Methimazole was used as internal standard. Limits of detection ranged between 7.0 and 611.7 mg·kg^−1^. Free amino acids have been used as chemical descriptors to differentiate between the ripening stages. Principal component analysis and linear discriminant analysis have been used as chemometric techniques, achieving complete differentiation between the ripening stages. Alanine, tyrosine, glutamine, proline, 2-aminobutanoic acid, cysteine, and valine were the most differentiating amino acids.

## 1. Introduction

Iberian dry-cured ham is a meat product manufactured following traditional methods [1]. It is highly appreciated by the consumers, and there is an increasing demand in the last years because of its good culinary qualities. Additionally, animal husbandry is done outdoors, improving the animal welfare, reducing the environmental impact, and protecting the ecosystem [2,3]. Iberian dry-cured ham has a preponderant role in the economies of several Spanish production areas, and various Protected Designations of Origin (P.D.O.) at the southwest of the country have been established, being P.D.O. “Los Pedroches” one of them [4]. Traditionally, the production of dry-cured ham consists of salting, postsalting, drying, and a final stage in a cellar. During the postsalting, drying, and cellar stages, ripening of the ham takes place. The time and duration of the postsalting, drying, and cellar stages vary depending on the type of dry-cured ham. In addition to lipolysis and oxidation, during ripening, endogenous enzymes degrade lipids and proteins to fatty and amino acids, which have an important influence on the flavor of dry-cured ham [5,6]. The time for the drying and cellar stages has an important influence on the quality of the final product. It takes from 23 months to 2–3 years for the highest-quality dry-cured hams [7]. A longer time of ripening leads to greater enzymatic degradation with an important effect on taste and flavor, producing a higher-quality dry-cured ham [8]. For this reason, the characterization of dry-cured ham and studies on their chemical evolution related to taste and flavor qualities are of great interest. In this way, various fractions have been studied, such as the volatile and the lipidic fractions of subcutaneous fat [9,10,11,12].

In the decade of the 1950s, amino acids have been determined by ion-exchange chromatography followed by post-column derivatization with ninhydrin and UV detection [13,14]. Reversed-phase high-performance liquid chromatography (HPLC) has been used for amino acid determination in the last thirty years, due to the flexibility of the technique and giving the possibility of using fluorescence detection and a higher sensitivity [15]. Analytical methods based on mass spectrometry (MS) and tandem MS (MS/MS) have also been reported for the determination of amino acids in foods [16,17]. In recent years, gas chromatography (GC) combined with flame ionization detector (FID) and/or mass spectrometer (MS) has been more commonly applied to the analysis of amino acids [18,19,20,21,22,23,24]. GC methods require a derivatization step to increase the volatility of the amino acids, being the methods based on the formation of silyl derivatives more commonly used [25,26]. 

Till now, the amino acids have not been considered for the differentiation of the ripening stage in the elaboration process of Iberian dry-cured ham. In this paper, the free L-amino acids alanine, 2-aminobutanoic acid, aspartic acid, cysteine, glutamine, glycine, histidine, hydroxyproline, isoleucine, leucine, lysine, methionine, phenylalanine, proline, serine, threonine, tyrosine, and valine have been determined by gas chromatography in Iberian dry-cured ham at postsalting, drying, and cellar ripening stages. Those amino acids have been used as chemical descriptors to differentiate between the three ripening stages. Principal component analysis (PCA) and linear discriminant analysis (LDA) have been used as pattern recognition methods.

## 2. Materials and Methods

### 2.1. Reagents and Standards

All solvents employed were HPLC-grade. N-Hexane and multisolvent TM HPLC ACS grade were purchased from VWR (Barcelona, Spain), and diethyl-ether, methanol, acetonitrile, and anhydrous pyridine were provided by Merck (Darmstadt, Germany). L-Amino acids standards: alanine, sarcosine, glycine, aminobutiric acid, β-alanine, valine, leucine, proline, isoleucine, serine, threonine, methionine, aspartic acid, hydroxyproline, cysteine, ornithine, citruline, arginine, phenyl alanine, glutamic acid, asparagine, homoarginine, lysine, histidine, glutamine, tyrosine, tryptophan, cistine, and hydroxytryptophan were supplied by Sigma Chemical Co. (St. Louis, MO, USA). Methimazole was used as internal standard and it was obtained from Sigma Chemical Co. (St. Louis, MO, USA). N,O-*bis*(trimethylsilyl)-trifluoroacetamide (BSTFA) from Sigma Chemical Co. (St. Louis, MO, USA) was used as derivatizing reagent. Other reagents were of analytical grade.

### 2.2. Samples and Sample Treatment

Hams from the protected designation of origin “Los Pedroches” were obtained from five Iberian 18-month-old pigs, fattened extensively with acorns, and pastured for 110 days prior to slaughter and processed in an industry for 26 months. In the following, the stages and the number of days since the beginning of the process are described. After the slaughter, hams were removed from the carcasses after 24 h and storage at 1 °C. Then they were placed in piles completely covered with marine salt with no contact between each other during one day for each kilogram of the ham. Next, the hams were hung at postsalting period during 90 days under controlled temperature and humidity, and then they were taken to a dryer at room temperature for 230 days. After this period, hams were left to mature during 400 days in a cellar. Consequently, the three steps considered in the process were postsalting, drying, and cellar. To cover the three steps of the process, samples were taken at 0, 60, 115, 180, 325, 440, 556, 653, and 783 days from the beginning of the postsalting step. The samples were taken from muscle, *biceps femoralis* and *cuadriceps femoralis,* and kept at −32 °C until analysis. 

### 2.3. Instrumentation and Gas Chromatography Analysis

A Varian 3800 gas chromatograph equipped with a fused silica capillary column of 30 m × 0.25 mm internal diameter coated with a 0.15 µm film thickness of DB-17HT (J&W Scientific, Albany, NY, USA) stationary phase, a flame ionization detector (FID), and a Varian 8400 automatic injector was used. The oven temperature was kept at 85 °C for 2 min, and it was then raised to 100 °C at a rate of 1 °C min^−1^. Next, it was then raised to 258 °C at a rate of 6 °C min^−1^. The injector temperature was kept at 290 °C, while the detector temperature was fixed at 310 °C. Hydrogen at a constant flow of 1.0 mL min^−1^ was used as carrier gas with a split ratio of 1:10. Air and hydrogen with flow rates of 300 and 30 mL min^−1^, respectively, were used for the detector, which had an auxiliary flow of 30 mL min^−1^ of nitrogen. 

GC–MS was applied to identify amino acids in the samples. A Varian CP3800 gas chromatograph coupled to a Saturn 2000 ion trap mass spectrometer (Varian, Palo Alto, CA, USA) equipped with a CP8400 autosampler was used. A DB-5MS (J&W Scientific, Albany, NY, USA) fused silica capillary column of 30 m × 0.25 mm i.d., coated with a 0.25 µm film thickness of DB-5MS stationary phase, was used. The oven temperature was initially kept at 90 °C for 2 min, and it was then raised to 120 °C at a rate of 2 °C min^−1^. Next, it was then raised to 258 °C at a rate of 5 °C min^−1^, followed by an isothermal period of 5 min at the latter temperature. The injector temperature was 290 °C. Hydrogen was used as carrier gas at 1.0 mL min^−1^ in constant flow mode and a split ratio of 1:20. The MS detector was operated in full scan mode from 25 to 650 amu at 1 scan/sec. Ion source and transfer line temperatures were kept 200 and 290 °C, respectively. The electron energy was 70 eV, a resolution of 1 and the emission current 10 µA were fixed. Dwell time and inter-channel delay were 0.08 s and 0.02 s, respectively. Varian Mass Spectrometry Workstation version 6.30 software (Varian, Palo Alto, CA, USA) was used for data acquisition and processing of the results.

The free amino acids content in the samples was determined according to the following procedure. Muscle samples of 0.5 g were cut up into small pieces and homogenized. Following, the samples were degreased with 3 × 10 mL of n-hexane-diethyl-ether (4:1 *v*/*v*) solvent extraction using a vortex agitator. Then, 1 mL of methanol containing 2.4 mg mL^−1^ of methimazole as internal standard was added and subsequently dried in a rotary evaporator at 30 °C under reduced pressure. The obtained residue was extracted with 3 × 5 mL of methanol-acetonitrile (1:1 *v*/*v*) using a vortex agitator. Of the solution obtained, 1 mL was filtered and next, evaporated to dryness in a rotary evaporator at 30 °C under reduced pressure. Acetonitrile (0.3 mL) and 0.3 mL of the derivatizing reagent (BSTFA) were added, and the mixture was heated at 80 °C during 30 min to obtain the trimethylsilyl derivatives. Of this solution, 1 µL was injected into the gas chromatograph. Triplicate analyses were performed.

### 2.4. Chemometrics

For chemometric calculations, a data matrix was prepared. The analyzed amino acids are used to describe the ham samples. Pattern recognition methods were applied to the data matrix, composed of 18 columns that correspond to the analyzed amino acids and 45 rows that are the ham samples. The data were analyzed using Statistica 8.0 software (Statsoft Inc., Tulsa, OK, USA).

Chemometrics is applied with several purposes. By one side, we try to visualize tendencies of the samples along the ripening process. On the other side, we are looking for appropriate classification rules to differentiate between postsalting, drying, and cellar stages. Additionally, information about the discriminant capacity of the variables can be obtained. 

Tendencies of the samples can be studied by using principal component analysis (PCA). PCA obtains new variables as linear combinations of the variables that, in this case, are the determined amino acids. The new variables are called principal components (PCs). There is no correlation between these PCs [27,28]. They are obtained in a sequential way, and each successive PC considers the remaining variability. The information provided by the determined variables is condensed in first PCs. Usually, the two first PCs, PC1 and PC2, account for an important part of the information, and plots using PC1 and PC2 as variables (scores plot) are very useful to visualize the trends of the data matrix. [29].

A classification rule was obtained by applying linear discriminant analysis (LDA). Calculations carried out with LDA produce discriminant functions (DFs) that are obtained as linear combinations of the variables which best separate the three considered ripening stages of the process. LDA is a hard modelling technique because the memberships of every sample and the number of classes or groups of samples have to be previously known. In this case, three classes, postsalting, drying, and cellar classes, are considered and two DFs have been calculated. 

## 3. Results and Discussion

### 3.1. Determination of Free Amino Acids by Gas Chromatography

Chromatographic analysis provides rapid and reliable separation of chemically similar compounds in complex food matrices. Dry-cured ham includes compounds with a wide range of polarities, some of them have low polarity, like lipids, and others are strongly polar, like amino acids [30]. GC is a high-resolution technique very useful to afford the analysis of this type of sample. Due to the low volatility, derivatization of free amino acids is performed for their analysis by GC. Several methods have been proposed in the literature. Some of them are based on multi-step procedures, which involve esterification of the carboxyl group followed by acylation of the remaining functional groups, and others are based on the formation of the silyl ethers [31]. Several derivatizing reagents were tested, including BSTFA, N,O-bis(trimethyldilyl)acetamide (TRI-SIL/BSA), and trimethyl-chlorosilane/hexamethyldisilazane/pyridine. The most effective derivatization was achieved when using BSTFA, while TRI-SIL/BSA and trimethyl-chlorosilane/hexamethyldisilazane/pyridine showed a very low efficiency. The use of a bulkier silylating group avoided the inconvenience of multiple derivative formation observed with some amino acids [31]. In order to improve the reaction efficiency and due to the acid hydrolysis reaction that takes place, small amounts of acetonitrile were added. Optimization of temperature and time of reaction was carried out, and the final optimal conditions were as follows: addition of 0.3 mL of acetonitrile and 0.3 mL of the derivatizing reagent (BSTFA) at 80 °C during 30 min. 

**Figure 1:**
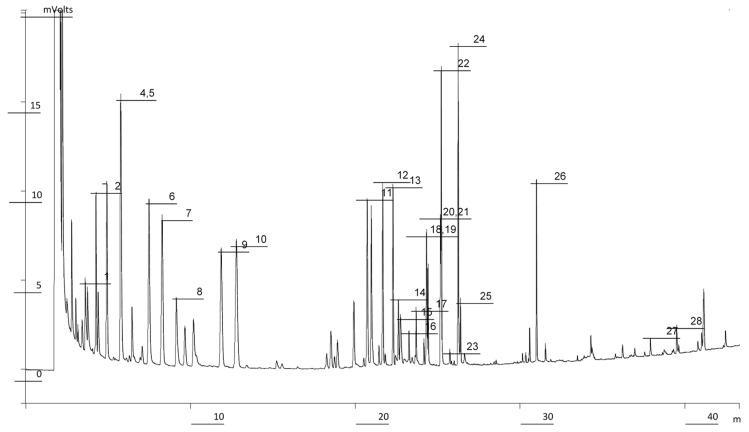
Chromatogram of amino acid standards obtained by GC–FID. Chromatographic conditions are included in paragraph 2.3. Peak numbers corresponding to amino acids appear in Table 1.

Several types of columns have been used to perform the analysis of amino acids by GC, some of them being low-polarity columns of methylpolysiloxane or silicone phases, methyl 5% phenylpolisiloxane, and others medium-polarity columns with methyl 50% phenyl polysiloxane [26,32]. In this work, a high temperature column with methyl 50% phenyl polysiloxane stationary phase DB-17HT has been used for the determination of the amino acids. Figure 1 shows a chromatogram of standards obtained using FID detector. The relative retention times of the amino acids to L-methionine are included in Table 1. In general, a good separation of amino acids was obtained. It is asserted that resolution (R) of two consecutive chromatographic peaks is complete if, at full width at half height of the smallest one, they do not overlap. Thus, analyzing in more detail the chromatogram of the amino acids in Figure 1, we can notice that complete separation was obtained for all the amino acids except for L-valine and L-β-alanine, L-glutamine and L-glutamic acid, L-histidine and L-homoarginine, with *R* values of −0.163, −0.059, and −0.054, respectively. 

**Figure 2:**
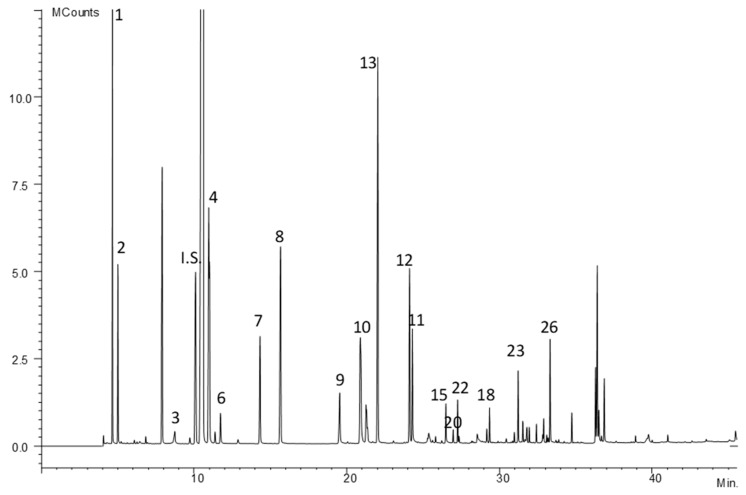
Chromatogram of amino acids in Iberian dry-cured ham obtained by GC–MS. Chromatographic conditions are included in paragraph 2.3. Peak numbers corresponding to amino acids appear in Table 2.

A total of eighteen free amino acids were identified in the Iberian dry-cured ham samples. Figure 2 shows the GC-ion trap-MS chromatogram profile in full scan mode of the trimethyl silyl ethers of the free amino acids fraction isolated according to the method proposed. The tentative assignment of the chromatographic peaks was done comparing the spectra with those from NIST 98 (National Institute of Standards and Technology, Gaithersburg, MD, USA) and WILEY 7 libraries and verified in every single case by standards. Table 2 shows the relative retention times to methimazole, the base peak and the molecular ion for these compounds. The identified amino acids present in Iberian dry-cured ham samples were L-alanine, L-2-aminobutanoic acid, L-aspartic acid, L-cysteine, L-glutamine, L-glycine, L-histidine, L-hydroxyproline, L-isoleucine, L-leucine, L-lysine, L-methionine, L-phenylalanine, L-proline, L-serine, L-threonine, L-tyrosine, and L-valine. These eighteen amino acids were quantified using methimazole as internal standard, considering that the relative response factor to free amino acids is close to the unit. 

Though there are many techniques available for the analysis of amino acids, the previous step of deproteinization is still one of the major problems. Peptides and proteins should be removed because they can interfere in the analysis and separation, as clogging the chromatographic column [33]. Precipitation with 5-sulphosalicylic acid, followed by centrifugation, ultrafiltration, and extraction are some of the most commonly used methods of deproteinization. In meat products, separation with organic solvents like methanol, dichloromethane, and chloroform has been used [23,33]. In this work, a previous step of solvent extraction using n-hexane-diethyl-ether has been performed for degreasing the samples. Then, a mixture of methanol-acetonitrile was used to extract the amino acids. 

For each amino acid, the calibration curves were obtained at the corresponding range of linearity. Each curve was prepared six times with a sample in which different amounts of every determined amino acid, at the levels 0, 80%, 100%, and 120%, were added. The calculated equations, area = slope × [mg kg^−1^] + intercept, are presented in Table 3. As it can be observed, a good correlation was obtained in all cases for a linear fit. The respective peak areas fitted a linear model within the indicate range shown in Table 3. The higher the wideness of linearity range, the more reliable the linear fit is. The slopes of the calibration lines for all amino acids ranged between 314.31 and 730.34, the highest value of this was for L-threonine, and the lowest for L-methionine. 

The trueness was assessed based on recovery assays that were carried out in the following way. A sample of Iberian dry-cured ham muscle was analyzed ten times by the proposed method. The obtained results for the different amino acids have been used as reference values. Then, different amounts of every determined amino acid at 80%, 100%, and 120% levels were added to the same sample, and ten replicates have been done for each case. The obtained results are shown in Table 4. As it can be seen, recoveries lie within the range 52–164% that can be considered acceptable values according the analyzed concentration [34] and consequently, trueness is significant. 

For the determination of repeatability, replicates were done on different days and in the same laboratory. Table 4 shows the obtained results. Relative standard deviations (RSD) range between 6.28% and 17.81%. These values of RSD are minor compared with the reference value derived from Horwitz equation. Therefore, the results for different amino acids indicate a good repeatability for the assay. 

The limits of detection (LOD) and limits of quantitation (LOQ) were obtained. The LOD of the method was determined considering a signal-to-noise ratio of 3 with reference to the background noise obtained from a blank sample. LOQ was calculated considering a signal-to-noise ratio of 10. Values of LOD and LOQ are shown in Table 4. The LOD obtained were between 7.0 and 611.74 mg kg^−1^, and LOQ ranged from 27.9 to 1779.9 mg kg^−1^. The lowest LOD and LOQ were for L-methionine, and the highest for L-isoleucine. 

**Figure 3:**
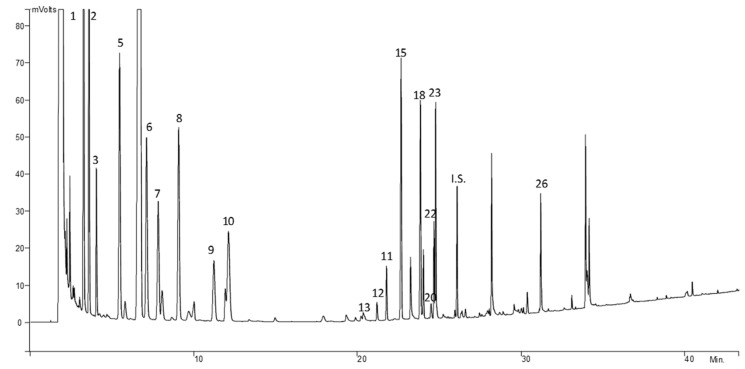
Chromatogram of amino acids in Iberian dry-cured ham obtained by GC–FID. Chromatographic conditions are included in Setcion 2.3. Peak numbers corresponding to amino acids appear in Table 2.

Figure 3 shows a GC–FID chromatogram of a sample of dry-cured ham. At the beginning of this amino acids chromatogram, there are some high peaks corresponding to free fatty acids, according to previously described in literature [26]. As it is shown in Figure 3, the free amino acids in Iberian ham muscle samples in higher amounts are L-alanine, L-glycine, L-valine, and L-proline, followed by L-leucine, L-cysteine, L-glutamine, and L-isoleucine. However, this fact disagrees with literature from other authors using a different technique [35], in which the Iberian ham, in its ripening end step, had higher amounts of the free amino acids L-glutamic acid, L-alanine, L-leucine, and L-glycine [35]. On the other hand, free amino acids in lesser amount were L-aspartic acid and L-methionine. Moreover, L-glutamic acid had not been detected [35].

### 3.2. Evolution of Free Amino Acids during Dry-Curing Process

**Figure 4:**
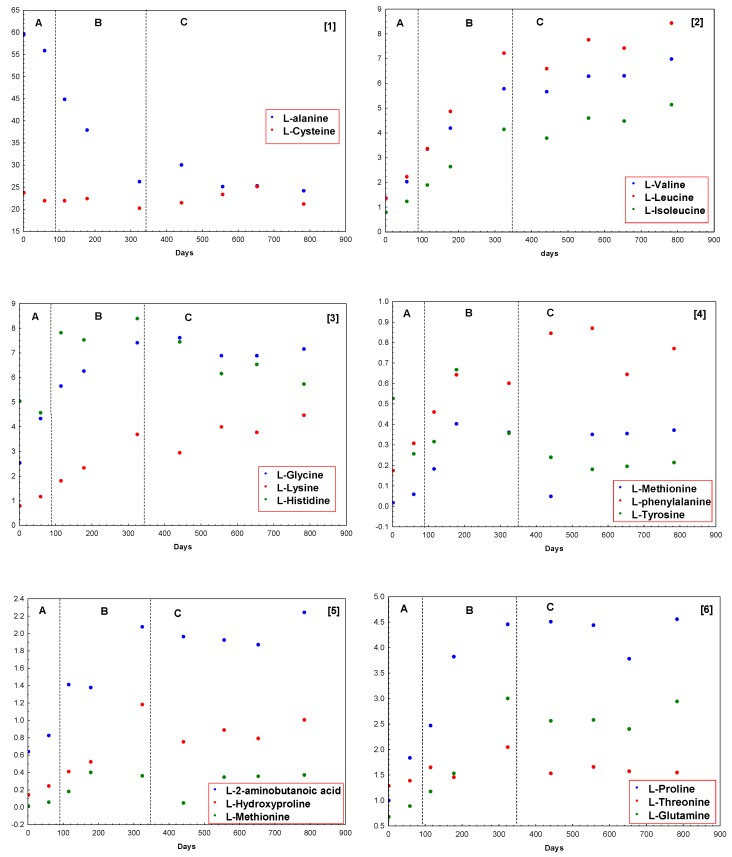
Variation of percentages of different amino acids (from 1 to 6) in Iberian dry-cured ham according to the ripening time.

Concentrations of amino acids in samples of Iberian dry-cured ham at different times of the ripening process have been determined. Samples at 1, 33, 64, 99, 180, 245, 309, 363, and 435 days were taken and analyzed according to the proposed GC–FID method. The sampling days account for the three considered ripening stages, postsalting, drying, and cellar. As it has been mentioned above, eighteen free amino acids have been detected in the samples throughout the ripening period of dry-cured Iberian ham. Results are included in Table 5. The total amount of free amino acids all along the process increases, which agrees with previous results described in the literature [35]. From a starting value of 6387.32 mg Kg^−1^ to a final one of 10320.55 mg kg^−1^, the following equation can be considered: [amino acid] = 5.7767 × (days) + 4714.6; *R*^2^ = 0.8034. Table 6 includes the variation of percentages of free amino acids according to the ripening time. In this way, an easier form to visualize the evolution of the amino acids in the samples is achieved. Figure 4 shows these percentages grouped for the different amino acids. As it can be seen in this figure, L-alanine is the main amino acid, with a significant decrease during postsalting and drying process, showing a maximum value of 59.56% of the total free amino acids fraction at the beginning of the process, and it stabilizes around 25.00% all along the cellar step (Figure 4(1)). The other main amino acid found in samples is L-cysteine, which remains fixed all along the process between 23.77% and 20.31%, as shown in Figure 4(1). L-lysine (Figure 4(2)), L-valine, L-leucine, and L-isoleucine (Figure 4(3)) increase all along the ripening process with a linear trend. On the other hand, the remainder components of this fraction suffer an increase that may be considered linear during postsalting and drying process to be fixed in cellar stage (Figure 4(3–6)). Only L-aspartic acid does not show, apparently, any trend during all the process.

### 3.3. Differentiation between Ripening Stages

**Figure 5:**
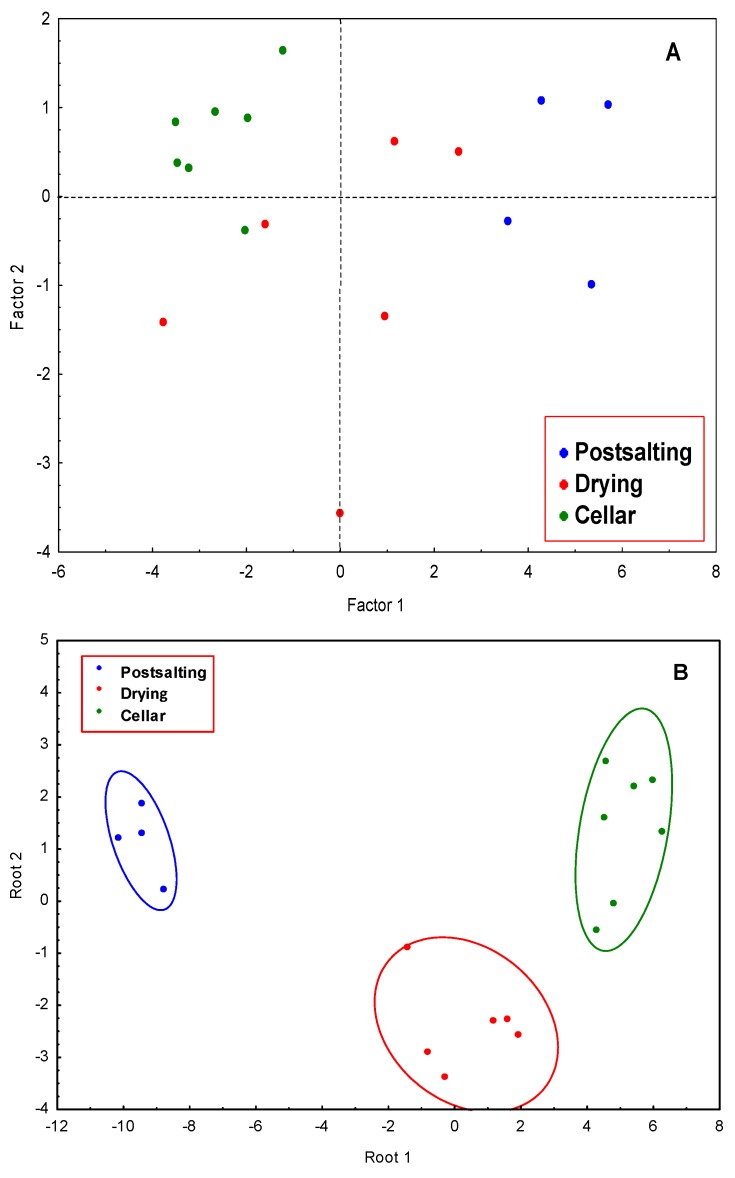
(**A**) Scores plot of Iberian dry-cured ham samples for the first PCs; blue, red, and green dots correspond to postsalting, drying, and cellar samples, respectively; (**B**) Scatter plot of the Iberian dry-cured ham samples in the plane of the two first DFs (discriminant functions).

To differentiate between the three ripening stages considered, postsalting, drying, and cellar, some chemometric calculations have been performed using the percentages of free amino acids obtained in the analysis of the samples. By applying PCA to the data matrix, two PCs were obtained. PC1 explains 69.08% of the variance, and PC2 9.66%, accounting these two PCs for 78.64% of the total variance. Figure 5A shows the scores plot considering the two first PCs. PC1 is strongly influenced by L-alanine, L-glycine, L-2-aminobutanoic acid, L-valine, L-leucine, L-isoleucine, L-proline, L-serine, L-aspartic acid, L-methionine, L-glutamine, L-phenylalanine, and L-lisine, and PC2 by L-cysteine, and L-histidine. The correlation between these variables is higher than 0.7. Though the samples corresponding to the different stages are not completely separated, some tendencies can be appreciated. Samples at postsalting stage are located in the scores plot at positive values of PC1, and those corresponding to cellar stage are situated at negative values of PC1. However, the samples corresponding to drying period appear spread between positive and negative values of PC1. An overlapping can be appreciated, and it could be explained considering this is a continuous process, being the first samples of drying mixed with those of postsalting and the last samples of drying mixed with the samples of cellar. This is an important consideration because in the process followed in processing plants, there are no clear criteria for transferring hams from drying to cellar stages, and the results obtained in this study can be very useful to optimize the elaboration process.

A classification rule to differentiate between the three stages was obtained by applying LDA. Using Wilks’ Lambda of 0.00532, two discriminant functions (Root 1 and 2) were obtained. Backward stepwise analysis retained the amino acids L-alanine, L-tyrosine, L-glutamine, L-proline, L-2-aminobutanoic acid, L-cysteine, and L-valine that can be considered the most discriminant variables. The classification functions are shown in Table 7. A complete separation between the three stages was obtained, denoting that the considered variables are powerful descriptors to differentiate between the samples from three dry-curing periods considered, postsalting, drying, and cellar.

## 4. Conclusions

By using GC–MS and GC–FID, eighteen free amino acids have been identified and determined in Iberian dry-cured ham at different ripening stages. Postsalting, drying, and cellar stages have been considered. During the ripening process, significant increasing of the amounts of all free amino acids, except L-alanine, was appreciated. To differentiate between the three ripening stages and using the free amino acids as chemical descriptors, PCA and LDA were applied. Using LDA, a total differentiation between the three ripening periods was obtained. The trends of total free amino acids in postsalting, drying, and cellar periods, respectively, can be used in order to predict the curing time.

## Figures and Tables

**Table 1:** Amino acids in a standard solution analyzed by GC–FID.

Peak	Amino Acid	T_rr_
1	L-Alanine	0.167
2	L-Glycine	0.191
3	L-2-Aminobutanoic acid	0.219
4	L-Valine	0.258
5	L-Alanine	0.259
6	L-Leucine	0.332
7	L-Isoleucine	0.369
8	L-Proline	0.429
9	L-Serine	0.533
10	L-Threonine	0.572
11	L-Hydroxyproline	0.934
12	L-Aspartic acid	0.972
13	L-Methionine	1.000
14	L-Arginine	1.009
15	L-Cysteine	1.020
16	L-Citrulline	1.031
17	L-Cystine	1.032
18	L-Glutamine	1.091
19	L-Glutamic acid	1.095
20	L-Histidine	1.127
21	L-Homoarginine	1.130
22	L-Phenylalanine	1.132
23	L-Lysine	1.136
24	L-Asparagine	1.186
25	L-Ornitine	1.203
26	L-Tyrosine	1.373
27	L-Tryptophan	1.659
28	L-Hydroxytryptophan	1.765

Compounds as trimethylsilyl derivatives; T_rr_ relative retention time to L-methionine. See Figure 1 for peak numbers.

**Table 2:** Amino acids identified in the Iberian dry-cured ham by GC–MS.

Peak	T_rr_	Amino Acid	Formula	M^+^	B.P.
1	0.211	L-Alanine	C_9_H_23_NO_2_Si_2_	233	116
2	0.227	L-Glycine	C_8_H_21_NO_2_Si_2_	219	102
3	0.396	L-2-aminobutanoic acid	C_10_H_25_NO_2_Si_2_	247	130
4	0.497	L-Valine	C_11_H_27_NO_2_Si_2_	261	144
6	0.532	L-Leucine	C_12_H_29_NO_2_Si_2_	275	158
7	0.711	L-Isoleucine	C_12_H_29_NO_2_Si_2_	275	158
8	0.650	L-Proline	C_11_H_25_NO_2_Si_2_	259	142
9	0.887	L-Serine	C_12_H_31_NO_3_Si_3_	321	73
10	0.948	L-Threonine	C_13_H_33_NO_3_Si_3_	335	73
13	1.000	L-Methionine	C_11_H_27_NO_2_SSi_2_	293	176
12	1.095	L-Aspartic acid	C_13_H_31_NO_4_Si_3_	349	73
11	1.103	L-Hydroxyproline	C_14_H_33_NO_3_Si_3_	347	229
15	1.202	L-Cysteine	C_12_H_31_NO_2_SSi_3_	337	219
22	1.237	L-Phenylalanine	C_15_H_27_NO_2_Si_2_	309	73
23	1.417	L-Lysine	C_15_H_38_N_2_O_2_Si_3_	362	84
18	1.332	L-Glutamine	C_14_H_34_N_2_O_3_Si_3_	362	156
20	1.495	L-Histidine	C_15_H_33_O_2_N_3_Si_3_	371	154
26	1.513	L-Tyrosine	C_18_H_35_NO_3_Si_3_	397	218

Compounds as trimethylsilyl derivatives; T_rr_, relative retention time to methimazole; M^+^, molecular ion; B.P., base peak; See Figure 2 for peak numbers.

**Table 3:** Calibration parameters for quantitative determination of amino acids.

Amino Acid	Slope	Intercept	*R* ^2^	Linearity Range *
L-Alanine	427.35	−145,386	0.9993	[0.00, 2310.35]
L-Glycine	499.7	−283,535	0.9999	[0.00, 1936.80]
L-2-Aminobutanoic acid	378.51	−22,077	0.9958	[0.00, 683.86]
L-Valine	441.26	−161,050	0.9999	[0.00, 1830.53]
L-Leucine	316.03	43,927	0.9643	[0.00, 1792.65]
L-Proline	346.04	−390.02	0.9969	[0.00, 1426.28]
L-Isoleucine	603.9	−362,852	0.9996	[0.00, 1143.00]
L-Serine	627.99	−159,368	0.9452	[0.00, 543.14]
L-Threonine	730.34	−293,682	0.9993	[0.00, 852.07]
L-Methionine	314.31	588.9	0.9804	[0.00, 35.07]
L-Aspartic acid	422.81	−5239.1	0.9965	[0.00, 66.43]
L-Hydroxyproline	464.86	−23,686	0.9898	[0.00, 209.95]
L-Cysteine	316.75	3075.1	0.9931	[0.00, 1017.84]
L-Phenylalanine	353.7	36.418	0.9998	[0.00, 434.64]
L-Glutamine	445.06	−38,852	0.9999	[0.00, 865.38]
L-Lysine	347.24	−5227.5	0.9840	[0.00, 578.92]
L-Histidine	358.7	−457.87	0.9991	[0.00, 606.50]
L-Tyrosine	343.04	−136.99	0.9995	[0.00, 532.76]

* mg kg^−1^.

**Table 4:** Repeatability, recovery, LOD (limits of detection), and LOQ (limits of quantitation) of amino acids analysis in Iberian dry-cured ham by GC–FID.

Amino Acid	Mean *	SD	RSD	Recovery	LOD *	LOQ *
L-Alanine	2336.9	151.8	6.5	101.36	457.2	1468.0
L-Glycine	1139.8	104.0	9.1	102.63	516.2	1521.0
L-2-aminobutanoic acid	358.0	59.6	16.7	113.05	55.5	160.3
L-Valine	920.2	80.4	8.7	121.31	322.7	913.9
L-Leucine	730.6	84.7	11.6	144.12	172.5	641.9
L-Proline	836.8	52.5	6.3	107.73	611.7	1779.9
L-Isoleucine	514.9	55.4	10.8	133.92	79.2	261.3
L-Serine	332.6	42.4	12.8	94.54	250.6	732.2
L-Threonine	417.9	39.0	9.3	113.02	308.5	1067.3
L-Methionine	17.1	3.0	17.5	164.13	7.0	27.9
L-Aspartic acid	64.1	11.2	17.4	52.29	32.0	77.8
L-Hydroxyproline	136.5	13.0	9.5	99.10	100.5	303.3
L-Cysteine	770.9	137.3	17.8	77.68	427.4	1484.7
L-Phenylalanine	158.4	19.3	12.2	157.30	96.9	262.1
L-Glutamine	575.0	38.7	6.7	91.34	362.2	1219.6
L-Lysine	461.0	29.5	6.4	77.83	231.1	735.3
L-Histidine	291.5	33.9	11.6	114.73	17.2	54.4
L-Tyrosine	258.3	12.7	4.9	131.43	196.1	845.4

* mg kg^−1^; average of the three spiking levels (80%, 100%, 120%); SD, standard deviation; RSD, relative standard deviation, RSD and Recovery in %.

**Table 5:** Variation of concentration * of amino acids according to the ripening time.

	Ripening Time (Days)								
Amino acid	1	33	64	99	180	245	309	363	435
L-Alanine	12,175.0	10,973.1	8392.8	6015.3	8610.5	5011.0	6349.8	4631.1	10,409.5
L-Glycine	512.8	922.9	914.9	967.2	2695.5	87.7	108.9	89.4	126.4
L-2-aminobutanoic acid	127.4	163.2	234.4	225.7	909.8	1655.6	1931.1	1115.2	2990.7
L-Valine	312.1	468.8	530.4	642.0	2262.4	471.9	528.5	308.8	957.9
L-Leucine	307.1	493.2	509.7	701.1	2748.9	1709.1	1890.9	957.5	2814.1
L-Isoleucine	166.0	272.3	290.4	400.3	1586.8	2164.6	2295.7	1035.5	3207.1
L-Proline	239.5	397.6	382.5	446.7	1657.8	1212.9	1379.1	607.9	1953.3
L-Serine	74.8	212.1	196.5	196.7	990.8	1142.1	1438.7	657.4	2014.2
L-Threonine	218.2	252.1	225.9	274.0	735.2	495.6	720.9	342.5	1086.8
L-Methionine	23.6	54.7	45.2	82.7	448.2	154.5	479.5	264.2	852.0
L-Aspartic acid	4545.8	4270.4	4317.4	4175.1	6602.8	296.9	271.3	97.5	360.3
L-Hydroxyproline	66.9	57.6	39.5	82.7	396.3	3650.9	6446.7	4515.1	8892.4
L-Cysteine	171.8	198.8	207.0	352.6	1264.5	691.4	799.8	450.0	1490.0
L-Phenylalanine	65.1	87.6	114.2	147.0	804.0	345.3	390.1	205.2	684.8
L-Lysine	44.2	44.6	38.8	77.3	204.3	203.9	42.0	53.8	357.8
L-Glutamine	155.9	256.7	251.9	340.6	1330.8	1027.9	1242.7	512.2	1510.4
L-Histidine	1448.6	1252.7	1376.7	1287.1	3420.0	1743.4	1622.3	1285.5	2645.8
L-Tyrosine	122.7	70.5	51.1	54.4	117.2	154.1	60.3	29.8	90.2
TOTAL	20,778.5	20,481.9	18,183.4	16,567.3	36,965.8	22,463.9	28,307.2	17,521.6	42,878.7

* mg kg^−1^.

**Table 6:** Variation of percentages (%) of amino acids according to the ripening time.

	Ripening Time (Days)								
Amino acid	1	33	64	99	180	245	309	363	435
L-Alanine	58.60	53.66	46.32	36.53	23.41	22.55	22.68	26.99	24.53
L-Glycine	2.47	4.51	5.05	5.87	7.33	0.39	0.39	0.52	0.30
L-2-aminobutanoic acid	0.61	0.80	1.29	1.37	2.47	7.45	6.90	6.50	7.05
L-Valine	1.50	2.29	2.93	3.90	6.15	2.12	1.89	1.80	2.26
L-Leucine	1.48	2.41	2.81	4.26	7.47	7.69	6.75	5.58	6.63
L-Isoleucine	0.80	1.33	1.60	2.43	4.31	9.74	8.20	6.03	7.56
L-Proline	1.15	1.94	2.11	2.71	4.51	5.46	4.93	3.54	4.60
L-Serine	0.36	1.04	1.08	1.19	2.69	5.14	5.14	3.83	4.75
L-Threonine	1.05	1.23	1.25	1.66	2.00	2.23	2.57	2.00	2.56
L-Methionine	0.11	0.27	0.25	0.50	1.22	0.70	1.71	1.54	2.01
L-Aspartic acid	21.88	20.88	23.83	25.35	17.95	1.34	0.97	0.57	0.85
L-Hydroxyproline	0.32	0.28	0.22	0.50	1.08	16.43	23.03	26.31	20.95
L-Cysteine	0.83	0.97	1.14	2.14	3.44	3.11	2.86	2.62	3.51
L-Phenylalanine	0.31	0.43	0.63	0.89	2.19	1.55	1.39	1.20	1.61
L-Lysine	0.21	0.22	0.21	0.47	0.56	0.92	0.15	0.31	0.84
L-Glutamine	0.75	1.26	1.39	2.07	3.62	4.63	4.44	2.99	3.56
L-Histidine	6.97	6.13	7.60	7.82	9.30	7.85	5.79	7.49	6.23
L-Tyrosine	0.59	0.34	0.28	0.33	0.32	0.69	0.22	0.17	0.21

**Table 7:** Results of the stepwise LDA.

Amino Acid	Postsalting ^#^	Drying ^#^	Cellar ^#^	F to Remove	*p*-Level
L-alanine	269.1	275.5	283.3	5.42201	0.032484
L-Tyrosine	251.4	265.3	271.1	4.42997	0.050692
L-Glutamine	−128.0	−173.5	−179.8	6.02760	0.025319
L-Proline	664.1	703.9	723.3	8.42128	0.010754
L-2-Aminobutanoic acid	1306.4	1398.7	1440.2	7.51229	0.014574
L-Cysteine	359.8	374.1	385.4	10.85224	0.005261
L-Valine	1410.9	1448.3	1493.2	7.12056	0.016739
Constant	−14584.7	−15553.3	−16467.7		

^#^*p* = 0.3333; *F* value, 2.8.
